# Data Centre Profile: The Provincial Health Data Centre of the Western Cape Province, South Africa

**DOI:** 10.23889/ijpds.v4i2.1143

**Published:** 2019-11-20

**Authors:** A Boulle, A Heekes, N Tiffin, M Smith, T Mutemaringa, N Zinyakatira, F Phelanyane, C Pienaar, K Buddiga, E Coetzee, R van Rooyen, R Dyers, N Fredericks, A Loff, L Shand, M Moodley, I de Vega, K Vallabhjee

**Affiliations:** 1 Department of Health, Provincial Government of the Western Cape, Cape Town, South Africa; 2 School of Public Health and Family Medicine, University of Cape Town, Cape Town, South Africa; 3 Division of Health Systems and Public Health, Department of Global Health, Faculty of Medicine and Health Sciences, Stellenbosch University, Cape Town, South Africa

## Abstract

**Introduction:**

The Western Cape Provincial Health Data Centre (PHDC) consolidates person-level clinical data across government services, leveraging sustained investments in patient registration systems, a unique identifier, and maturation of administrative and clinical digital health systems.

**Objectives:**

The PHDC supports clinical care directly through tools for clinicians which integrate patient data or identify patients in need of interventions, and indirectly through supporting operational and epidemiological analyses.

**Methods:**

The PHDC is housed entirely within government. Data are processed from a range of source systems, usually daily, through distinct harmonisation and curation, beneficiation, and reporting processes. Linkage is predominantly through the unique identifier which doubles as a pervasive folder number, augmented by other identifiers. Further data processing includes triangulation of multiple data sources for enumerating health conditions, with assignment of certainty levels for each enumeration. Outputs include patient-specific email alerts, a web-based consolidated patient clinical viewing platform, filterable line-listings of patients with specific conditions and associated characteristics and outcomes, management reports and dashboards, and data releases in response to operational and research data requests. Strict architectural, administrative and governance processes ensure privacy protection.

**Results:**

In the past decade 8 million unique people are recorded as having sought healthcare in the provincial public sector health services, with current utilisation at 15 million attendances or admissions a year. Cross-sectional enumeration of health conditions includes over 430 000 people with HIV, 500 000 with hypertension, 235 000 with diabetes. Annually 110 000 pregnancies and 54 000 patients with tuberculosis are enumerated. Over 50 data requests are processed each year for internal and external requesters in accordance with data request and release governance processes.

**Conclusions:**

The single consolidated environment for person-level health data in the Western Cape has created new opportunities for supporting patient care, while improving the governance around access to and release of sensitive patient data.

## Background

**Figure 1: Western Cape Provincial Health Data Centre - high level architecture:**
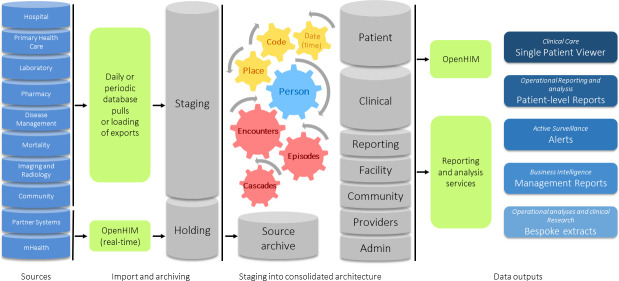
Data from various source systems (left) are acquired on either a daily or periodic basis and imported into a Staging database. Data from mHealth and partner systems are imported into a Holding database real-time through an information mediator (OpenHIM). The data in Holding are moved to Staging during the daily load. A copy of all source data is archived after import to enable the data structures to be re-built without repeating the extract from the source systems. In Staging, data are prepared for the consolidated architecture by ensuring each record has a date/time stamp, that each record can be assigned a place (e.g. health facility or community outreach region) and that, where required, the data can be mapped to a common coding system (such as ATC codes for drug data or LOINC codes for lab data). Each record is mapped to a person in the PMI using a linkage engine that compares the identifiers received from the source system to the patient identifiers in the PMI. Once data are linked and cleaned data beneficiation occurs by means of inferring health conditions (episodes), inferring health service contacts (encounters) and by pre-building of patient-level cascade reports. The prepared data are imported into the consolidated architecture which stores Patient and Clinical data in separate databases linked only by an internal unique patient key. The architecture also consists of databases for Facilities, Community-based data, providers, Administrative data, and pre-built data for Reports. Above the database layer there is the information mediator as well as reporting and analysis services from which various outputs are available to meet the 5 primary objectives: (1) Clinical Care (Single Patient Viewer), (2) Operational reporting and epidemiological analysis (provided through patient-level reports accessible on SharePoint), (3) Active Surveillance (provided through patient-level alerts and cascade reports that are updated daily, (4) Business intelligence (provided through management reports on service level utilisation), and (5) Operational analyses and clinical research (provided through bespoke extracts).

The Western Cape (WC) Provincial Department of Health (PDoH) has over the past two decades gradually implemented patient administration systems in all fixed public-sector facilities, which share a unique health identifier or patient master index (PMI) [1]. Increasing availability of patient-level data linkable by PMI resulted in new opportunities for data to improve care and services. This led to the establishment of a Provincial Health Data Centre (PHDC) in late 2015, intended primarily to impact service delivery, directly through clinical tools actionable at patient-level, supporting continuity and quality of care, and indirectly through health system intelligence actionable at organisational unit level (Figure 1).

In the envisaged PHDC model, aggregate and management reporting are a natural by-product of clinical and administrative systems, without the need for parallel systems to record and aggregate data, as has been the norm for District Health Management Information Systems in many African settings, including in South Africa [2, 3]. The data further support in-depth epidemiological analyses, whether conducted in-house as part of routine public health surveillance, or in support of the research enterprise, through well governed data releases with strict adherence to ethical and patient protection prescripts (Figure 1).

Two key features differentiate the PHDC from traditional data linkage centres [4]. Firstly, the same environment services both clinical needs based on linked named patient data, and analytic and research needs based on linked anonymised data. This is made possible by the single-provider nature of the government health system. Secondly, enumerating health conditions requires a complex approach which leverages a range of data sources to infer health conditions, in contrast to health systems with well-coded claims-based data or a culture of high-quality clinical coding. This inference approach categorises outputs based on certainty, such that only inferences with high certainty are used for clinical tools, while also enabling exploration of potential misclassification bias in epidemiological analyses based on more expansive enumeration which includes this certainty score.

As an organisational entity, the PHDC is entirely contained and managed within the provincial government health services. It is located at the PDoH head office alongside core strategic functions including planning, epidemiology and surveillance, health research stewardship, information technology and information management. Since inception, PHDC functioning has been supported by seconded staff from health system improvement and surveillance projects which required an integrated, person-level data environment. Future recurrent core costs are however planned to be part of routine government budgeting and funding. The shift in focus from aggregate to individuated data has required the creation of new posts, building institutionalised data competence to work with large volumes of complex person-level health data.

## Approach

The PHDC was incrementally established, beginning in 2012 with a data harmonisation project to test the feasibility and completeness of data linkage on the PMI [5]. This progressed to formally establishing the data centre in order to build the internal capacity to work with the emergent data resources.

The manner in which the PHDC was established, and choices around technology and architecture, were pragmatic. This was based on the availability of resources and technology, the available data and associated methods of access, skills within the team, and the desire to implement standard approaches to interoperability and data warehousing in health care.

### Population setting

The Western Cape Province is one of nine provinces in South Africa, and has close to 7 million inhabitants, three quarters of whom use the public-sector services. For many high burden health conditions such as HIV and tuberculosis, the vast majority of patients use the public sector for all or most of their care. The government health services operate as a single provider, with salaried staff on fixed remuneration [6]. The burden of disease in the Western Cape mirrors that in South Africa, with infections such as HIV and tuberculosis together with violence and injury, being the largest contributors to premature mortality and years of life lost [7]. The burden of disease in the setting is often referred to as the quadruple burden of disease, with the addition of maternal and child health issues, and a rapidly increasing burden of non-communicable disease [8]. Key health metrics for this population (2012) include life expectancy at birth of 66 years, infant mortality of 19 per 1000, and maternal mortality ratio of 79 per 100,000 [7].

There are an estimated 15-million patient contacts annually at 52 hospitals and 272 primary care clinics managed by the PDoH, and 82 clinics managed by the City of Cape Town municipality. In some larger hospitals, patient registration has been digitalised for over 40 years. These legacy patient registration data were onboarded with the progressive implementation of Clinicom™, a single shared hospital patient administration system, across all hospitals, starting in 1998. From 2007, patient registration systems in primary care clinics have progressively also accessed the central PMI. Complete coverage of all fixed facilities with compliant patient registration systems was recently achieved, with a total of over 17 million historical registrations of whom - accounting for duplicates - an estimated 8 million have been active in the past decade.

### Operating model

The PHDC is a repository model, with database repositories for facilities, patients, clinical data, providers, and households and community members. Data for clinical domains are either pragmatic abstractions of more complete datasets residing in source systems, for example medicine dispensing software databases, or comprise metadata and clinical summaries relating to image repositories stored on source systems, such as radiology images and formatted laboratory reports. For some domains, such as referrals between fixed health services and services provided by community health workers, the PHDC fulfils the functions of a Health Information Exchange, through bidirectional standards-based interoperability.

### Architecture and information technology

Data are received through multiple mechanisms: uploaded via linked database server access; pushed to source-specific holding databases on the PHDC infrastructure; loaded through a health information mediator to a holding database [9]; fetched from remote drives or ftp server locations; pushed to PHDC source-specific ftp sites; and manually extracted and uploaded on a schedule. Where needed, limited python-based parsing harmonises large file-based sources prior to data import.

The PHDC evolved on the Provincial Government enterprise Microsoft SQL Server™ platform to ensure support and sustainability within the context of the standard tools available in a large government bureaucracy. A full application lifecycle management stack has been provisioned for the PHDC on virtualised servers by the transversal information technology providers for the provincial government, consuming 30TB of storage spread across 8 virtual servers. While compute provision is currently conservative, it can be increased on an as-needed basis, especially as the environment is expected to migrate to cloud and platform-as-a-service technologies.

### Governance, legislation and management

The PHDC operates under the direct management of the PDoH, with governance through line-management of staff, and joint accountability to oversight committees for information technology, data governance and health research.

Overarching health (National Health Act 61 of 2003), personal privacy (Protection of Personal Information Act 4 of 2013), mental health (Mental Health Care Act 17 of 2002), vital registration (Births and Deaths Registration Act 51 of 1992), information access (Promotion of Access to Information Act 2 of 2000), and medicines (Medicines and Related Substances Controls Act 101 of 1965) legislation are the most important for guiding conduct around health information and the operations of a data centre which processes named and anonymised patient data.

Where the PHDC data directly inform patient care, the same prescripts apply as for data in any source system or contributing electronic medical record. Broadly, the operational sharing of named data is only permissible where it facilitates direct delivery of health services to the patient in keeping with the original reason for which the identifying data were provided.

There is strict governance on the release of data for research purposes, with each province required to have a Provincial Health Research Committee (PHRC), and every health research project registered on the National Health Research Database. All research data requests are formally approved by the PHRC given there is ethical and government approval of the protocol, and consistency of the request with the approved protocol. National legislation and health research ethical standards require explicit patient consent for the release of named data, except in highly exceptional circumstances deemed by an independent ethics committee and health authorities to be justifiable in order to protect population health.

### Consent model

As the PHDC is part of the operational infrastructure which supports direct patient care, patients do not explicitly consent to the inclusion of their data in the PHDC. As discussed above, approval for access to named data is only provided where this is in line with clinical need and function, or as part of an ethically approved research data release, which almost invariably requires patient consent.

Whereas national guidance does not require patient consent for the release of anonymised data, local stakeholders have encouraged the Province to explicitly inform patients how their data are shared for clinical use, and that their anonymised data may be used in analyses for health service improvement or accessed by researchers. The PDoH aspires to further provide the option to patients to opt out of the use of their anonymised data, pending maturation of administrative systems to support this.

### Privacy by design

On data take-on, the named patient data are separated from the attribute or clinical data and stored in separate databases, linked by an internal anonymous key. This linkage key, although it cannot be used to identify a patient, is in turn never released without being differentially perturbed for each dataset, preventing future cross-linkage of independently generated datasets.

Database access by developers and analysts is tightly managed according to IT governance standards, with access to the patient database containing patient identifiers restricted to only those instances where it is absolutely required to have such access, and subject to specific approvals and undertakings. Whereas most analyses can be conducted on de-identified data safely accessed through reporting tools, core analysts doing primary development typically would also not require or have access to the database with patient identifiers. All analysts and developers sign strict confidentiality agreements, and agree to adhere to good practice standard-operating-procedures (SOPs), which minimise inadvertent risks of inappropriate patient identification. There is automatic logging of any direct or application-driven query which accesses the patient database, ensuring accountability. Details of all patients who have data included in data releases are logged to facilitate any future requests under access-to-personal-information legislation.

The database infrastructure is located on the government intranet, subject to the full security and IT governance applicable to the whole environment.

All data are released according to a strict SOP. Identifiers, when provided, are always released separately to attribute data. All released datasets are encrypted and password-protected, with passwords communicated by different mechanisms to the file transfer method. Anonymisation where required involves the issuing of a study-specific anonymised unique patient identifier, perturbation of an index date (e.g. date of birth) with additional dates reflected in relation to the index date, grouping ages into categories wherever possible, limiting the provision of facility data, and avoiding provision of fine-grained geographic information.

#### Data linkage

Data linkage comprises linkage within the PMI in order to identify all possible PMI-duplicates, as well as the linkage of all attribute data from source systems to the PMI. Initially a deterministic linkage algorithm was developed to assign a certainty level to matches, for purposes of simplicity and transparency. Matches based on canonical identifiers (e.g. civil identifier or hospital folder number) with some corroboration confer higher certainty than those based on other identifiers. Extensive use is made of text-edit-distance fuzzy comparisons based on reviews of the best performing fuzzy comparators in the context [10]. A fully probabilistic score is also available through a SQL implementation of standard probabilistic record linkage approaches [11], but is not yet productionised, pending validation against a curated linked dataset, which will also be used to assess the current linkage efficacy. An explicit blacklist and whitelist cater for where it is known that the linkage engine will repeatedly incorrectly match or not match records. Linkages are stored in a lookup table, and linkage chains are assessed and revised through a transitive closure approach. A history of all links between identifiers from the sources and the PMI is stored, from which prior successful links can be rapidly leveraged, hugely improving computational efficiency.

As household members enumerated by community-based services are not necessarily expected to be clients of the health service and may not appear on the PMI, the community database is searched for retrospective linkages and historical household-derived data, with all new additions to the PMI.

### Data linkage keys

The patient registration systems of the hospitals and primary-care clinics all issue the same 9-digit numeric folder number, the last digit of which is a modulus-10 checksum. The folder number is intended to be common across the platform for a given patient. This is printed on barcoded labels, such that most clinical stationery has a label with this number affixed. As a result, when data are received from source systems, linkage to the PMI is frequently aided by the availability of a folder number.

### Data sources

The hospital information system, Clinicom™, is a core data source as it currently provides the PMI-functionality, and thereby includes all patients whether accessing hospital or primary care facilities. This system, based on the Cache™ hierarchical database, is queried daily for new additions to the PMI, hospital admissions and outpatient visits, and associated administrative data including admission and discharge dates, procedure and diagnostic codes, and discharge summaries. Key additional data sources include laboratory and pharmacy data, and data from bespoke databases designed to assist in program management for high priority conditions, such as electronic registers for HIV and tuberculosis. The laboratory data are of particular value in the continuity-of-care assessments and outcome evaluation for chronic disease management. South Africa has a national laboratory service in which digitised pathology results are available from one source for the entire public sector [12]. While not all medicine dispensing is digitised, where these data are available in hospitals, large clinics and from pre-packaged dispensing, they are invaluable given the high proportion of patients who receive pharmacy items with each facility visit. A full list of data sources with details of take-on, coding and volume is included (supplementary file 1).

An important current gap is vital registration data. A change to the death notification forms in 2013 reserved cause-of-death details for access by the national statistical agency, which is prohibited from releasing identifiable data. This prevents the PDoH from currently accessing definitive cause-of-death information from the authoritative source [13].

### Data processing

The activities of the PHDC are broadly characterised as harmonisation and curation, data beneficiation, and data provision. The onboarding of source data harmonises each source into the consolidated PHDC architecture. This includes curation in which each data point is mapped to a person (the PMI linkage previously described), location, and standard ontology, such as ICD10 codes for diagnoses, ATC codes for medicines, and where possible LOINC codes for diagnostic tests.

Beneficiation involves using all available data to infer health service contacts (encounters), health conditions (episodes) and health outcomes for conditions (cascades). The generation of a master list of patient encounters, ranging from household visits, to ambulance transfers, to primary care and hospital visits and admissions, is invaluable for tracking the care and utilisation trajectories of patients. For conditions of public health importance such as HIV and tuberculosis where patients out-of-care drive presentation with advanced disease as well as transmission, this is especially valuable. For many primary-care encounters visits are inferred from laboratory and pharmacy records to compensate for incomplete clerical systems.

**Figure 2: Inference approach for health conditions:**
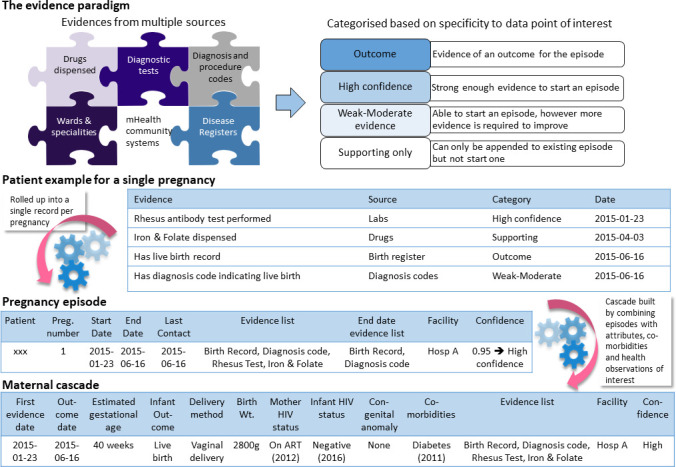
Episodes are inferred from multiple data sources which carry evidence of a particular health condition. Evidences are categorised as Outcome, High Confidence, Weak-Moderate or Supporting and assigned a score. The example of pregnancy is presented in the figure showing how a high confidence episode is “rolled-up” or combined for an individual with a Rhesus antibody test (performed almost exclusively on pregnant women in the public sector), iron and folate dispensing (a supporting evidence) an ICD10 code (weak evidence due to unreliable clinical coding) and a live birth record in a register (outcome). From the pregnancy episode a maternal cascade is built containing details about the pregnancy, information about the infant, HIV status and transmission information, as well as co-morbidities. The cascade also links dynamically to the Single Patient Viewer so that the electronic health record and/or contact details of individuals requiring follow-up can be easily accessed.

The limited coverage and quality of diagnosis coding has resulted in the use of multiple data sources for inferring health conditions. All data points (or selected combinations) are assessed for evidence that could indicate a health condition, for example antenatal blood tests denoting pregnancy or hypoglycaemic drugs denoting diabetes. Evidences are weighted and combined to create a summative score for the enumeration of the health condition (Figure 2), adapted from bioinformatics methods for summarising protein-protein associations [14]. This allows confidence in the inferred condition to be strengthened based on multiple sources of evidence, and for evidence of ongoing care to be inferred from otherwise non-specific evidence. For example, iron and folate supplementation would not be considered as sufficient evidence of pregnancy, but in known pregnant women would be considered to be evidence of ongoing pregnancy-associated care. Using common health conditions as illustrative, province-wide, for mid-2019, the PHDC enumerates 430,000 patients with HIV who have been in care in the previous 2 years, excluding those known to have died. Similarly, 235,000 patients with diabetes and 500,000 with hypertension are enumerated. For episodic conditions, the PHDC currently enumerates 110,000 pregnancies and 54,000 tuberculosis infections annually.

The sensitivity and specificity of the inference approach, especially for episodes designated as being of high certainty, is yet to be formally evaluated, in part due to the absence of obvious gold-standard datasets. As data are requested for patients with known health conditions enrolled in research studies or service-based local registries which were not previously incorporated as evidences, it is anticipated that the performance of the approach will be able to be continuously assessed against these previously unseen curated datasets.

Episodes of health conditions are enriched into cascades, which contain information about further characterisation (e.g. drug-resistant infections), comorbidities (e.g. tuberculosis in HIV-infected patients), retention in care (e.g. continued chronic drug dispensing), assessment of control (e.g. viral load in HIV-infected patients), and outcome (e.g. microbiological cure for tuberculosis). The cascades are essentially virtual and continuously-updated cohorts from which a range of alerts and reports can be created. Patients with a health condition can be listed for a jurisdiction, and filtered by a range of utilisation, disease characteristics or outcome criteria.

### Data access

The PHDC simultaneously services clinical, operational, management and research needs, and data access differs by purpose. Clinical access is provisioned through integrated clinical viewing of a single patient’s data, and access to named line listings of patients requiring specific interventions. Clinicians can apply for access to the associated web-based tools, which where possible are restricted to patients attending the facilities in which a clinician works. Clinicians sign undertakings committing to appropriate use for mandated care provision only.

For access by managers and clinical governance custodians to line-listing reports in which patient identifying details are hidden or obfuscated, data are still considered sensitive and users undertake to treat as such. The most important risks are potential re-identification when facilities and dates of service access and location, and clinical details are accessible.

Requests for data extracts are differentiated by provider requests aligned with their clinical or operational functions, sometimes including justification for named data, and external research requests, subject to the standard research approval processes described earlier.

To date it has not been necessary to provide safe-haven (locked-down, on-premises) access, as all data requests have been able to be fulfilled by data centre staff with anonymisation as required. Around 50 data releases are currently approved and provided per year split evenly between operational and research requests.

### Noteworthy outputs

The inclusion of disparate *data sources* in the PHDC is demonstrated by the onboarding of pregnancy data from a national health promotion program in which woman sign up for gestational-age specific phone-based text messages [15].

**Figure 3: Web-based clinical view (“Single Patient Viewer”) graphical integration example:**
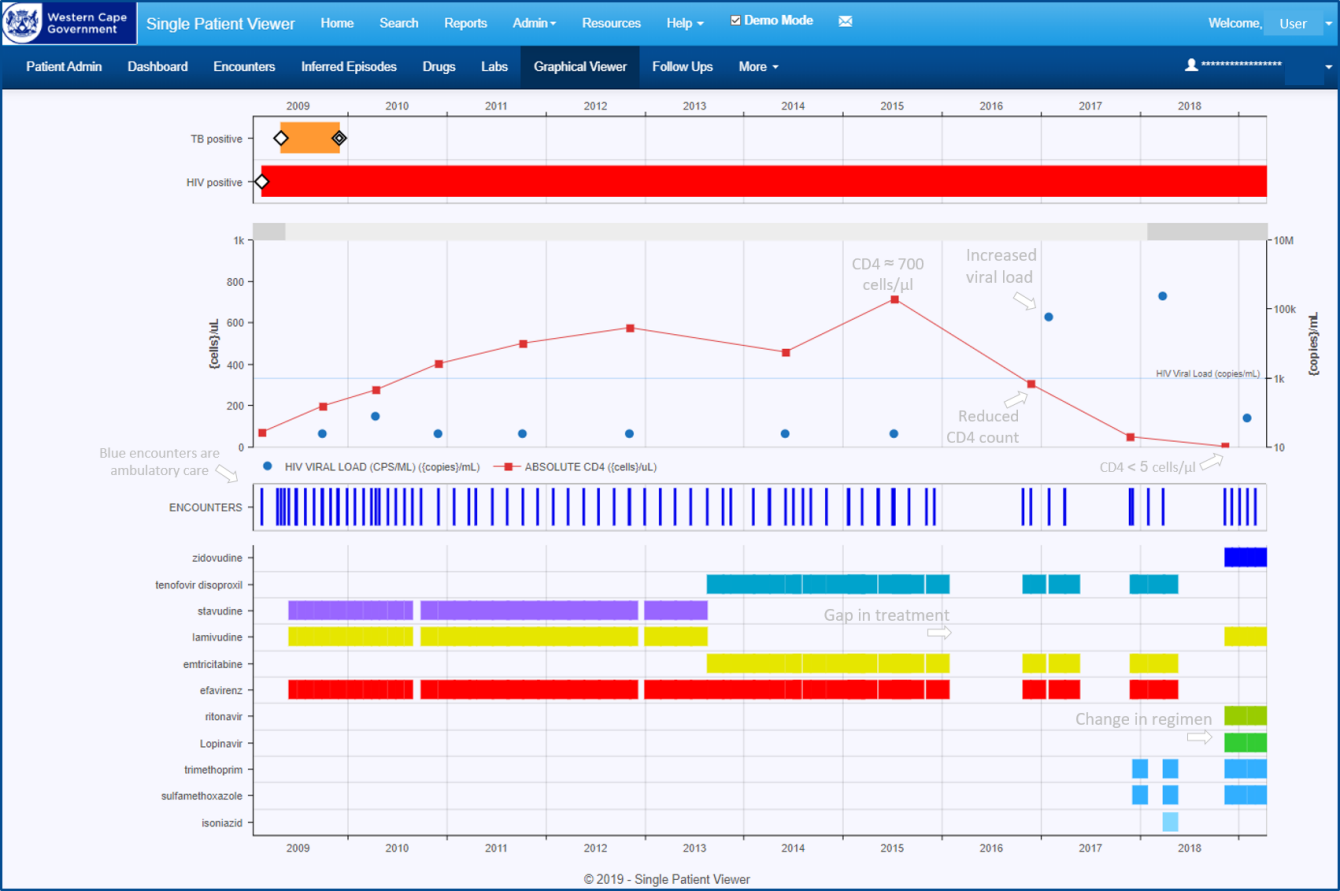
Clinical data for a given patient are consolidated longitudinally in the “Single Patient Viewer”, a web-based tool for clinician-use. A graphical viewer cartoons the enumerated health conditions (orange and red in the top pane). Clicking on one of these plots laboratory tests, health service encounters, and medicines of relevance to the condition. In this example, of an HIV-infected patient presenting with very advanced disease (CD4 count of <5 cells/μl), numerous treatment interruptions and missed opportunities to change a failing treatment regimen contributed to a 3-year decline in immunological status.

In terms of *clinical use*, a Single-Patient-Viewer (SPV) has been developed, which is a prototype for how these data could be used in a web-based electronic health record or portal. The tool integrates clinical data for a single patient both longitudinally and cross-sectionally, in tabular and graphical views (Figure 3), to assist clinicians in rapid information discovery. Currently around 100 clinicians (mostly doctors and clinical nurse practitioners) are using and testing the portal in an extended pilot phase, either for direct clinical care provision in both hospital and ambulatory settings, or as part of clinical audit activities.

*Alerts* are already widely used by laboratory services on identification of infections, but can be enriched by the PHDC with non-laboratory clinical data. It has been postulated that if health systems had the ability to be alerted each time a person with previous confirmed, possible or exposure to selected drug-resistant pathogens (such as Carbapenem-resistant Enterobacteriaceae [CRE]), the incidence of hospital-acquired drug resistance could be reduced [16]. The PHDC has recently implemented such an alert in response to a CRE outbreak, sent by email each morning to infection prevention and control officers at participating institutions, and as a pop-up alert on the SPV clinical viewer. The uptake and impact of this and other alerts has not been formally evaluated.

The first *report* to be accessed by a range of service providers was one which linked laboratory information on abnormal cervical cytology, to details of access to diagnostic and therapeutic colposcopy services in gynaecological follow-up clinics. This provides actionable individuated data on patients who require active follow-up, integrating data province-wide more efficiently than the historical paper-based registers.

Finally, some of the fulfilled *data requests* have facilitated population-wide research, such as exploring reasons for the ongoing burden of advanced HIV disease in spite of universal availability of treatment [17], based on anonymised linked individuated data on HIV patients from the time of their first evidence of HIV through to treatment, follow-up and immunologic and virologic assessment.

## Discussion

The WC PHDC is a logical consolidation of data resources, made possible through the sustained investment by the government health services over many years in digitising patient registration, implementing a unique identifier, achieving full coverage with core administrative systems in hospitals and clinics, and maturing a range of administrative and clinical digital solutions. There are a number of learnings related to the specific context.

### Generalisability of the model

The data sources available to the Western Cape are either common nationally, such as the laboratory and disease register data [12, 18], or have comparable alternatives in other provinces, including a national unique identifier which is now almost universally implemented in primary care [19]. Over time similar data consolidation should be possible in other health jurisdictions in the country and region.

### Shift from aggregate to individuated data

The shift in the region from aggregate to individuated data being at the core of health department information systems is likely a threshold effect. At a certain point the paper-based systems used historically to collect aggregate data elements start being digitised for efficiency and accuracy, and it increasingly makes sense for these to be derived from routine administrative or clinical digital processes, rather than recorded and digitised in parallel. As the number of digital systems proliferate and reporting requires access to cross-facility and cross-platform clinical data, information tools derived from pervasive consolidated environments start to have advantages over reporting from single-solution systems.

### Human capacity is the major investment and asset

One of the main benefits from formally establishing the PHDC has been developing the staff capacity to work with the large volumes of data resulting from this shift, which requires data science skills which have not generally been catered for on the PDoH staff establishment. The initial PHDC staff came from varied backgrounds including bioinformatics and demography and, in future, it is anticipated that graduates from nascent data science programmes will find the PHDC a fulfilling work environment, and be adaptable to changes in the digital health and corporate information technology landscapes.

### Dealing with incomplete and unreliable data

The inference approach based on different data sources and combinations of data points, and categorisation of the inference certainty, has allowed for enumeration to be more complete sooner than would have been possible if relying only on diagnostic coding or bespoke registers, and earlier use and iteration of data tools and outputs. This triangulation has proved critical for overcoming concerns about the data quality of any single source, and for extending enumeration to include previously unrecognised cases.

### Initial focus on operational rather than analytical requirements

The PHDC focus on supporting clinical care first and foremost, and deriving analytic value on the back of validated clinical data outputs, is critical for building trust that it is not an extractive exercise for the research community or for agency reporting. This contrasts with many data linkage exercises conducted for the purposes of estimating key program metrics, but which do not benefit from iterative improvements which derive from direct operational use of tools, and which are unable to directly impact the care of individual patients. All the requirements of case-based surveillance, proposed for example by some global agencies as a panacea for information gaps in the HIV response in high-burden settings [20], can frequently be met from appropriate consolidated data environments such as the PHDC, which simultaneously also directly support clinical care. This is due to linked data on case-detection, laboratory assessments, treatment access and outcomes being available from the single consolidated source in a timely manner, as described above for advanced HIV [17], obviating the need for a parallel disease-specific case-based surveillance system.

### Risks and benefits related to data consolidation in a single environment

Whereas there were some concerns about the patient protection risks being introduced by the consolidation of existing data resources in one environment, it appears that the ability to standardise processes and governance around data access have rather improved practice and reduced risks. The single environment has also assisted with resourcing the PHDC, as many system-improvement and operational research projects have substantial data components. Using funding from these to support and extend a consolidated data environment contributed to establishing the PHDC, and could be the catalyst for other similar jurisdictions to establish new forms of data competence.

## Conclusion and future developments

It is anticipated that the PHDC will continue to benefit from further improvements in source systems, including the introduction of electronic medical records. This will result in a shift in effort for the PHDC from data harmonisation to greater analytic support. Whereas the PHDC has focussed initially on clinical data, planned consolidation with existing business intelligence projects working with cost and provider data will create opportunities for data tools and outputs to assist with efficiency and quality initiatives. It is further envisaged that intra-government mechanisms will emerge for the safe and responsible sharing of data across departments, consented or through defined privacy-preserving solutions, in support of cross-sectoral and whole-of-society government initiatives.

## 


