# Supplementary material for: Data Centre Profile: The Provincial Health Data Centre of the Western Cape Province, South Africa
Source: Int J Popul Data Sci. 2019 Nov 20;4(2):1143. doi: 10.23889/ijpds.v4i2.1143 (PMC7482518; doi:10.23889/ijpds.v4i2.1143)
Supplement: Supplementary file 1 [file ijpds-04-1143-s001.pdf]

Table 1. Data Sources incorporated into the PHDC

Name	Category	Description	Period	Weekday volume	Records to date	Frequency	Method
Clinicom	Hospital	Clinicom hospital information system<br>The hospital information system in all 52 hospitals in the province which also provides the enterprise patient master index (PMI)	1994-	1600 new patients added to PMI<br>1700 new Admissions<br>18000 new outpatient visits for 12500 patients	9.4 million admissions<br>70.8 million outpatient visits	Daily	Linked Server
PHCIS	Primary care	Primary Health Care Information System<br>Used for patient administration and routine information collection in the 272 Western Cape Provincial primary care clinics, and as a disease register for HIV and TB in an increasing number of facilities.	2007-	30000 visits (unique by patient and facility)	44 million primary care visits	Daily	Holding database
PREHMIS	Primary care	Patient Registration and Health Management Information System<br>Used for patient administration and routine information collection in the 82 City of Cape Town primary care clinics	2015-	11000 visits (unique by patient and facility)	6 million primary care visits	Daily	FTP (internal)
NHLS	Laboratory	National Health Laboratory Service<br>Used for tracking and managing laboratory tests in all public health facilities in South Africa	2007-	177000 test result lines for 8600 patients	500 million records	Daily	FTP (external)
JAC	Pharmacy	JAC Pharmacy System<br>A centralised medicine management and dispensing system used in 44 hospitals and 65 primary care facilities in the Western Cape Province.	2010-	77000 items dispensed for 20000 patients	102 million items dispensed	Daily	Linked Server
CDU	Pharmacy	Chronic Dispensing Unit<br>An automated warehouse pharmacy which pre-packages and dispatches medication for stable chronic patients.	2012-	42000 items dispensed for 8000 patients	91 million items dispensed	Monthly	Manual retrieval
TIER.Net	Disease Management	Three Interlinked Electronic Registers<br>Off-line electronic register for monitoring HIV treatment in use in over 100 clinics, and into which data are imported from other HIV register systems	2001-	4300 new treatment visits<br>180 new patients registered	650,000 patient/facility combinations	Quarterly	Linked Server to central database into which dispatches are loaded from each site quarterly.

**ETR.Net**	Disease Management	**Electronic Tuberculosis Register**<br>The standalone electronic register for tuberculosis programme monitoring, being decommissioned during 2019. Pages from the paper tuberculosis register are sent to sub-district offices for capture into ETR.Net.	2001-	122 new patients started on TB treatment	820,000 registrations on ETR	Quarterly	Linked Server
**EDRWeb**	Disease Management	**Electronic Drug-restistant Tuberculosis Register**<br>Web-based information system for treatment of drug-resistant tuberculosis patients	2007-	50 new drug resistant TB patients started on treatment	43,000 drug resistant TB patients started on treatment	Weekly	Manual retrieval
**SINJANI**	Aggregate reporting	**Standard Information Jointly Assembled by Networked Infrastructure** (DHIS equivalent)<br>Web-based portal for collecting and reporting aggregate information in the Western Cape Department of Health, feeding into the national District Health Information System. The system also collects discrete sets of patient-level data such as maternal mortality data	2000-	1 or fewer new maternal deaths	885 maternal deaths	Daily	Linked Server
**EMS**	Emergency Transport	**Emergency medical services**<br>Call management and ambulance dispatching system for emergencies, with incident and limited clinical data variously available.	2014-	2300 calls logged	3.4 million calls	Periodically	FTP (internal)
**HealthNet**	Patient Transport	A system used for booking transport of patients from their home to a health care facility	2015-	1000 calls logged	620,000 calls	Periodically	FTP (internal)
**HECTIS**	Emergency Centre	**Health and Emergency Centre Tracking Information System**<br>A web-based interface for managing Emergency Centres that enables real-time tracking of patients from arrival to the Emergency Centre to Discharge, currently implemented at six hospitals in the province	2017-	400 EC visits	300,000 EC visits	Daily	Linked Server
**ECCR**	Hospital	**Electronic Continuity of Care Record**<br>A web-based platform for capturing discharge summaries with improved clinical coding	2016-	650 discharge summaries	390,000 discharge summaries	Daily	Linked Server
**Catch and Match**	Community	**Catch and Match community health worker application**<br>A mobile application used by Community Health Care Workers to assist screening of community members and to refer patients for follow up at health care facilities	2015-	60 new household registrations	10,000 households and 40,000 individuals	Hourly	Holding database

MomConnect	Maternal health	**MomConnect pregnancy health promotion solution**<br>A National Department of Health initiative to support maternal health through cell phone-based technologies	2014-	90 new registrations on MomConnect	120,000	Daily	FTP (internal)
Hello Doctor	Tele-triage	**Hello Doctor tele-consultation service**<br>A tele-triage system that allows patients to speak to a doctor telephonically and proceed directly to the pharmacy for over-the-counter medications	2017-	20 calls logged per day	7,500 calls logged	Monthly	Manual retrieval
PPIP	Mortality	**Perinatal Problem Identification System**.<br>An application developed by the Medical Research Council used in most paediatric departments to record adverse perinatal outcomes	2000-	2 new deaths per day	42,000 infant deaths recorded	Periodically	Manual retrieval
BOD	Mortality	**Burdern of Disease surveillance system**<br>Cause-of-death data from copied of official death certificates received from the Department of Home Affairs	2010-2013	N/A	126,000 deaths recorded	No longer receiving	-
ChIP	Mortality	**Child Healthcare Problem Identification Programme.**<br>A mortality audit tool designed specifically for infants and children (up to 18 years) with the aim to use reviews based on the data collected to improve the quality of care that sick children receive in the health system	-	-	-	Data transfer agreement in progress	-
WCBTS	Blood transfusions	**Western Cape Blood Transfusion Services**<br>Details of blood products ordered by public sector facilities	-	-	-	Data transfer agreement in progress	-
PACS	Radiology	**Picture Archiving and Communication System**<br>Details of radiology imaging from both PACS systems and associated radiology information systems (RIS) where implemented	-	-	-	Data transfer agreement in progress	-

*Daily volume shown is the approximate average volume of new records received per day on weekdays (Monday-Friday) from 2018-01-01 to 2019-05-31
